# Virtual 3D environments as composition and performance spaces^*^

**DOI:** 10.1080/09298215.2019.1703013

**Published:** 2019-12-20

**Authors:** Marko Ciciliani

**Affiliations:** Institute of Electronic Music and Acoustics, University of Music and Performing Arts Graz, Graz, Austria

**Keywords:** Game-based composition, virtual performance, intermedia performance

## Abstract

In the present text, I will reflect upon the composition of works that use virtual 3D environments in which the performers are represented as interacting avatars. In particular, I will explain the musical possibilities offered by this work with virtual environments, detailing the effects that the design of virtual topologies has on the interactive sound sources and sound events contained in these topologies. I will focus particularly upon how performers can musically influence said effects by navigating with the avatars acting within the topology.

## Introduction

My own experiences in this field were gained as part of the artistic research project GAPPP,^1^ which analyses the artistic potential of computer game elements in the context of audiovisual compositions.^2^ As a point of entry, I will start by investigating basic aspects of musical work with space and movement using the example of Tom Johnson’s composition *Nine Bells*. I will then use my own composition *Kilgore* (Ciciliani, n.d.), which was created as part of GAPPP, as a case study for my more detailed examination of this topic. After analysing general aspects that play an important role in compositional work with 3D environments, I will embark upon a discussion of questions concerning the representation of performers in the virtual space. Following a discussion of 3D environments’ analogies to instruments and scores, I will finally investigate a range of concrete issues and methods related to audiovisual composition using virtual spaces. As mentioned above, this investigation emerged from artistic research, which implies that knowledge and understanding is primarily gathered through artistic practice (Lüneburg, 2018). Although it is common in artistic research to use additional methods that enable the researchers to take a step outside in order to get a more objective perspective on the research phenomenon (Borgdorff, 2012), it is still widely accepted that the core of artistic research remains subjective. As a consequence, it has also become more common to formulate publications in first-person style, which I also consider to be the appropriate form for this text.

## Previous applications of virtual 3D space in concert-based works

Since a few years there has been a remarkable increase in the use of game engines for musical purposes. Softwares such as Unity (Unity Technologies) or Unreal Engine (Epic Games) nowadays offer easy access to designing interactive 3D environments, which only few years earlier posed significantly more challenging technical difficulties. Apart from a more general trend in contemporary composition to expand musical practices to various other media (Ciciliani, 2017), it seems plausible to attribute the strongly increased popularity of those softwares to their relative ease of use, and – last but not least – low cost.

In the history of media art there are various examples of projects in virtual 3D spaces that show a strong commitment to musical aspects, such as *Osmose* (1995) by Char Davies, *q3apd* (2002/03) by Julian Oliver and Steven Pickles, or *Fijuu* and *Fijuu2* by the same authors (Oliver & Pickles, 2007). However, in this summary I will mainly consider the use of 3D space in works based on performances in concert settings, since this is the focus of this chapter.

Although it is described as a graphical programming environment rather than an artistically employed 3D space in a concert setting, *The Audicle* can be regarded an interesting predecessor. It was developed by Cook and Wang (2004) as an editor, compiler, virtual machine, and debugger. What is more significant in this context is that it includes a carefully and aesthetically designed 3D environment that visualises computational processes, and that responds to real-time programming. It offers an aesthetic spatial experience in order to guide design decisions and make processes more comprehensible.

Composer and researcher Robert Hamilton published extensively on his work with game engines and virtual 3D space. Already in 2007 he investigated possibilities of tracking avatars in 3D space and using streamed data to control musical events (|Hamilton, 2007, 2008). Since, he also produced several remarkable works that employ virtual 3D space in concert settings. Especially worth mentioning are works he created in collaboration with Chris Platz such as *ECHO::Canyon* (Hamilton, 2014) and *Carillon* (Hamilton & Platz, 2016). More recently he has focussed on building virtual instruments that are played by musicians wearing VR-headsets, as in his virtual reality string-quartet *Trois Machins a la Grâce Aimante* (Hamilton, 2019). This work, however, does not offer navigation in a 3D environment.

In 2007 the NOVARS research centre was established at the University of Manchester, UK, which specialises in Electroacoustic Composition, Performance and Sound-Art (Climent, Berezan, & Davison, 2008). The centre was run from the start by Ricardo Climent who began to work with virtual 3D environments in the following year with his installation *Ho*. In the wake of this project, Climent composed a series of concert works based on virtual 3D space, starting with *Xi* in 2012 (Climent, n.d.). Furthermore, the work with immersive space became one of the core research areas of NOVARS which led to various PhD projects based on 3D space and game engines, such as Jose Ignacio Pecino Rodriguez dissertation (Pecino Rodriguez, 2015) who also published previously on this field of research (Pecino Rodriguez, 2014).

It is interesting to note that works using 3D environments have recently also found their way into competitions for composition that in their past mainly focussed on instrumental music. Composer and media artist Remi Siu was one of five finalists of the prestigious GAUDEAMUS award for young composers, held in Utrecht, The Netherlands, in September 2019. He entered the competition with *new notations* (Siu, n.d.) a multiplayer 3D environment that was performed by four musicians of the Nadar Ensemble. It thus seems safe to say that after one and a half decades of experimentation in the niches of mainly academic electronic music, by today, the composition with 3D environments has found its way into the mainstream of contemporary music.

## Composing with space and movement

Usually, the concept of ‘composing with space’ is understood as the purposeful positioning or movement of a sound event in a plane or three-dimensional space. My reflections are also concerned with the distribution of sound events in space, but here one important feature is that this spatial configuration can be experienced in a virtual space through performers’ movements. This section of the text will examine the interaction between the spatial configuration of a 3D space, the distribution of sound sources within that space, and the kinds of movements carried out by the actors. I will start with an analysis of Tom Johnson’s composition *Nine Bells* (1979).

Even though this piece makes no use whatsoever of virtuality or other technologies, it is a vivid illustration of some of the basic principles of composing with space and movement. The only sources of sound in this work, which lasts for just under an hour, are nine bells. They are positioned evenly in three regular rows of three bells on a square area measuring just under 4 × 4 metres. The composition is made up of nine parts. In each of them, the performer moves around the bells over and over on precisely defined trajectories, striking them according to specific defined patterns. Each part uses a different movement pattern, which then characterises the musical event. The melodic material is formed by the individual movement patterns, which make certain sequences of pitches possible and exclude others. The movement pattern likewise determines the time intervals between the strikes and thus the rhythmic sequence. It is crucial that the performers move at a constant speed, so that covering a certain distance always takes the same amount of time. Accordingly, it takes longer to reach bells that are further apart than those positioned directly next to one another.

**Figure 1.**
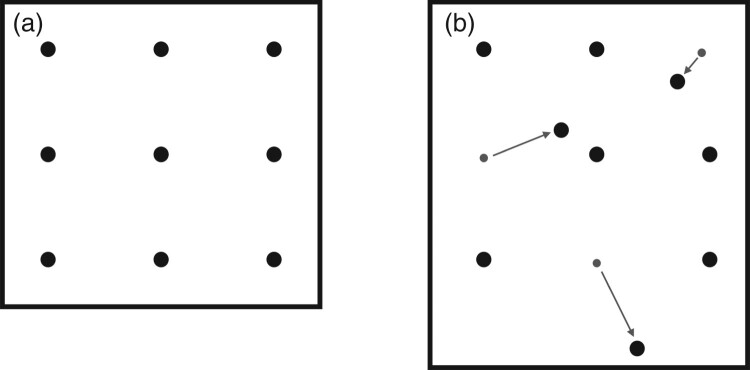
(a and b) On the left we see the original position of the nine bells in *Nine Bells*, on which the first computer simulation is based (a). The figure on the right shows the changed positions of three of the nine bells (b). These positions were used in the second computer simulation.

*Nine Bells* illustrates the principles of composing with space and movement in transparent and concentrated form. The same principles – the interaction of the distribution of the sound sources in the space on the one hand and the performers’ movement on the other – are also characteristic of working in virtual 3D spaces. If one aspect changes, this has a direct impact on the other, too, with consequences for the music. If, for example, the position of two bells were exchanged, but the sequence of notes retained, this would lead to a change in the movement pattern and thus also to a change in the time intervals. If we were to replace the regular, symmetrical distribution of the bells with an irregular pattern while retaining the same movement pattern, this would lead to different time intervals. I illustrated the latter case using two computer simulations that are available for viewing online. The first example shows an accelerated simulation of ‘First Bell’, the first part of *Nine Bells*, in a virtual three-dimensional space. The second example shows the same piece, but here the position of three of the bells has been altered. The resulting change in the intervals at which the bells are struck markedly changes the piece’s basic musical character.^3^ Figure 1(a,b) shows the changed positions of the bells.

At first, the difference between the two versions appears quite straightforward. By changing the distances between the bells while retaining the same movement pattern, the time intervals between the strikes change. However, it is worth taking a closer look at the musical differences: the even distribution of the bells endows the original version with a sense of calm that is already evident in the piece’s initial phase, in which only the middle bell is struck at comparatively large intervals. This temporal regularity is conveyed not just on an auditory, but also on a visual level, as in this phase the avatar is already circling four of the nine bells.

The timing of the asymmetrical version could be described as more unsettled or nervous, as the calming equal proportions of the original have been lost. At the same time, the asymmetrical version could also be regarded as more elastic, as the time intervals are expanded and compressed. These expansions and compressions repeatedly result in melodic conglomerations, giving rise to a different sense of melodic emphasis than in the regular original. For example, certain situations take on the emphasis of an upbeat, which was not perceptible as such in the regular version. In my view, the spatial repositioning of the bells has far-reaching musical consequences.

**Figure 2.**
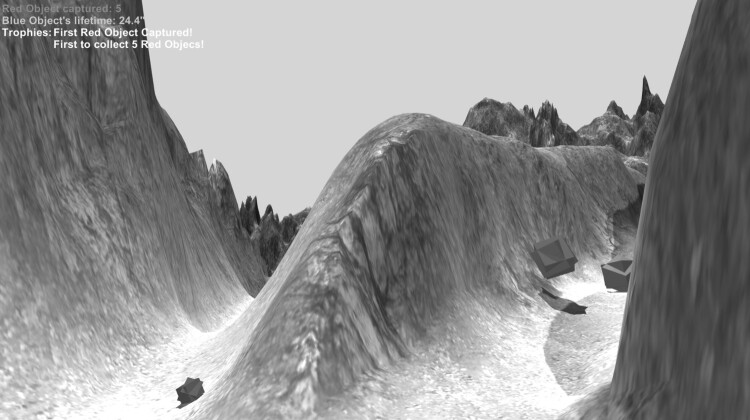
The performers’ perspective while they move through *Kilgore’s* virtual environment and interact with sound-producing objects.

In the following, I will examine to which extent these observations on the reciprocal effect of the positions of the sound sources and the movement patterns in *Nine Bells* can be applied to a virtual 3D space. My composition *Kilgore* (2018) will serve as a point of comparison. *Kilgore* is a composition lasting just under half an hour for two performers. In certain parts of the piece the performers play instruments, while in others they navigate in a virtual 3D space using game controllers. In this virtual space, they carry out actions that have both a direct and an indirect musical impact upon the composition. These actions create the work as a whole, and it is thus possible to say that the virtual 3D space is also the composition’s performance space. Figure 2 shows what the space looks like from the performers’ perspective.

**Figure 3.**
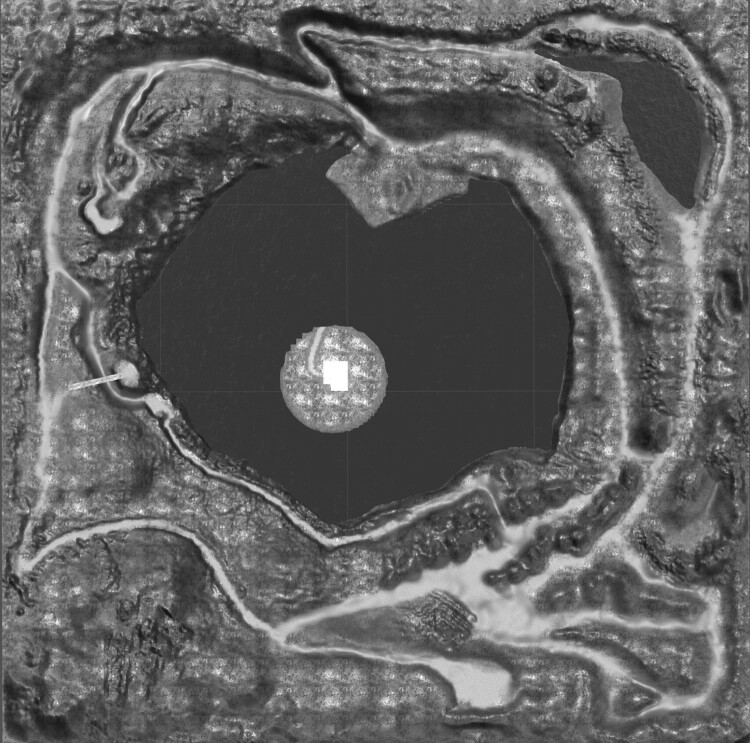
Cartographic view of the entire virtual space, which measures 500 × 500 virtual metres in total.

Comparing *Kilgore’s* performance situation with that of *Nine Bells*, I first have to point out that in *Kilgore* – unlike in *Nine Bells* – the performers can move around freely using their avatars. There is no set speed of movement, nor are there defined trajectories. Movement is shaped by the environment and the paths it contains. As can be seen in Figures 2 and 3, I designed the 3D environment as a mountainous landscape with different pathways, some of which have been literally carved into the terrain. If we now imagine that various musical affordances are distributed within this environment, we can see that, in musical terms, the connecting pathways and separating obstacles define sound sequences and time relations, even though the latter are not precisely defined as rhythmic values, as in *Nine Bells*, because the performers are able to move around freely.

While the bells in *Nine Bells* are distributed across a space of less than 4 × 4 metres, in virtual space we are naturally not confined by the practical limitations of actual performance spaces. In *Kilgore*, I worked with a landscape measuring 500 × 500 virtual metres. But what happens if we assume – analogously to the bells in *Nine Bells* – that the sound zones in *Kilgore* are distributed evenly and differ in the quality of their sound, and then reposition these zones? This would necessarily lead to changes that affect the piece’s large-scale musical structure. That which manifests as a variation in rhythm and melodic character in *Nine Bells* here becomes a change in formal structure.

We can thus deduce two fundamental insights from the work with movement patterns and sound sources distributed in space, which I will summarise here:

1. The spatial distribution of sound sources shapes a composition’s time factor. Certain combinations of sounds become more likely than others and are presented as more obvious. If the distances are shorter, as in the case of *Nine Bells*, the effects affect musical details such as rhythmic configuration and melodic sequence. On a larger time scale, the specific distribution shapes the piece’s formal structure.
2. The design of trajectories is directly linked to the previous point. In contrast to the precisely defined movement patterns in *Nine Bells*, when navigating in 3D environments it is customary to allow the actors to move freely within the space. Accordingly, we cannot predict which sounds will occur exactly when and in which combination. Nevertheless, the topology’s design determines whether certain spatial connections and sequences of sound are facilitated and others made more difficult. Thus we can assume that each decision concerning the design of a 3D environment, such as the inclusion of obstacles and passages, will have indirect or direct musical consequences.

Depending on our perspective, a 3D environment and the sound-generating object it contains can thus be seen either as an *instrument* or a *score*. I will return to this comparison in the next section but one, where I will describe an example in which the direct link between the landscape’s design and the associated musical consequences is evident.

## Representing the performers in the virtual space

As mentioned above, a performance of *Kilgore* is created in a concert situation in which two performers act on a real stage. At the same time, the virtual three-dimensional environments in which the performers are represented are shown on two projection surfaces.

The game engine Unity has been used to design the 3D environments. The two performers are running two instances of the same environment as standalone applications, although their start locations in the virtual environments are different. All sounds are produced in the software SuperCollider. Data streams with information on the movement and actions of the avatars and the locations of sound producing objects in the environments are exchanged between the two Unity instances and SuperCollider by using OpenSoundControl (Wright & Freed, 1997). While there are a number of different sonic scenarios in the piece, movements of the avatars always produce continuous sounds when moving in the horizontal plain and percussive sounds when jumping.

The performers explore their virtual environments from a *first person perspective*. While they themselves cannot be seen in the virtual world as 3D models, their individual perspective and movements mean that they can be identified. I refer to these representations as ‘avatars’. While this term is frequently used to designate a person’s digitally rendered visible form in a virtual space, I draw on the more general mythological concept of the avatar being the instantiation of a person’s essence into a physical body (Scarborough & Bailenson, 2014). In so doing, I emphasise the presence in another world rather than the question of visual appearance. This justifies my use of the term, even though the form itself remains invisible in the *first person perspective*. Accordingly, a major part of the performance is realised in the 3D space by the performers, who move within this space and interact with various sound functionalities via their avatars. Thus the virtual space also functions as a performance space.

In order to make it possible for the performers to experience being present in the virtual space, in *Kilgore* I took the simulation of a realistic situation as my starting point for the piece’s visual and the acoustic design. In concrete terms, this means that aspects such as navigation, gravitation and acoustics behave similarly to the way they do in reality – an approach that is also standard when designing computer games in 3D spaces. As far as the acoustic design is concerned, the sounds perceived correspond to the performers’ positions in the virtual environment, a phenomenon that Michel Chion describes as ‘subjective point of audition’ in the context of film (1990). In the present case, this subjective point of audition is based upon the implementation of the following three elementary aspects:

1. The amplitude of a sound source reduces square to the distance from it.
2. The sound source’s position in the stereo image corresponds to the performer’s orientation in the virtual environment. This means that if a sound source is located to a person’s left, they will hear it from that direction. By using a setup of speakers that expands vertically, the virtual sound source’s position on the vertical axes could also be represented accordingly.
3. If one turns one’s back to the sound object in the virtual space, a subtle filter is applied, which on the one hand schematically simulates the effect of the external ear and on the other hand accords sounds whose virtual source is not seen lesser acoustic presence.

Starting from this position creates the preconditions for a familiar acoustic experience, which in turn makes it possible for the performers to experience a so-called *virtual presence*, the experience of ‘being there’ (Riva & Waterworth, 2014). This means that the performers identify with their avatars and experience their virtual surroundings as their primary environment, interacting with and orienting themselves towards them. The precondition for this orientation is the ability to distinguish between a self and an environment that does not belong to that self (Waterworth & Waterworth, 2014). If we want to enable the performers to experience virtuality as a place into which they are intensely embedded, it is important to design its digital realisation – also referred to as *perceptually seductive technology* (Waterworth, 2001) – in a way that enables the experience of presence. Parallels to the experience of the real world form the starting point for this.

**Figure 4.**
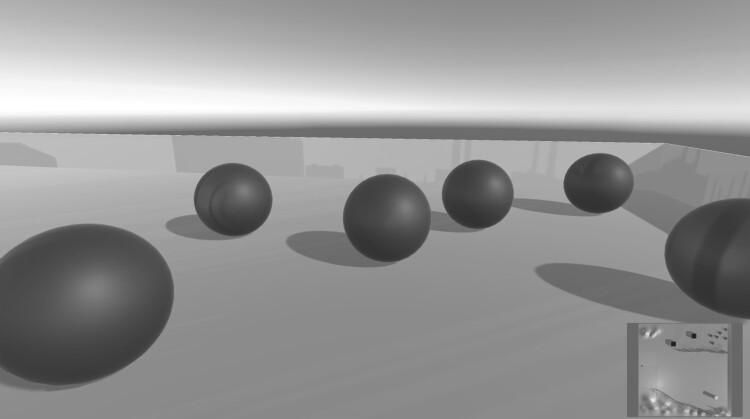
Examples of a model with six interactive, moveable balls producing different static pitches. Here, generating data that also includes the distances between the balls themselves could provide interesting results.

Interestingly, it is also possible to maintain the illusion of presence in a virtual space even if individual analogies to reality are abandoned: ‘It has been known for some time that it is possible for virtual reality to achieve a kind of ‘sensory rearrangement’ resulting in modified experiences of one's own body’ (Waterworth & Waterworth, 2014, p. 595). This is also referred to as ‘maximal binding’, ‘[which] implies that in cyberspace anything can be combined with anything and made to “adhere”’ (Novack, 1992). This is highly interesting and has hardly been investigated in regard to musical scenarios thus far. In an experimental setup (cf. Figure 4), for example, I replaced the third of the abovementioned elementary points, the filtering of sound when turning away, with a manipulation of pitch. This means that in this particular case, all sound sources located behind the avatar were transposed. As the sound sources in this model produced static pitches, rotating around one’s own axis in the virtual space resulted in a variation of the harmonic situation.

In my opinion, it is important to note that not only is the virtual presence still maintained in the case of such manipulations, even though a behaviour is introduced that no longer corresponds to reality, but also that such manipulations can also be dealt with intuitively as long as the context as a whole perpetuates the basic feeling of ‘being there’. In the text’s final section, I will investigate the technical aspects of this kind of virtually embodied steering of parameters in greater detail.

## The 3D environment as score and instrument

I would now like to discuss more closely the comparison of 3D environments with scores and instruments mentioned at the beginning of this text.

Calling an assortment of, for example, nine bells an instrument would normally not be questioned. Tom Johnson’s decision in *Nine Bells* to position the nine bells at a distance from one another does not change the bells’ sound potential, but it does shape the way they can be played in a specific manner. This means that the bells’ basic function as musical instruments remains intact. When a 3D environment is designed with the aim of offering a certain arrangement of possible sounds, this environment can thus be understood as an instrument.

By contrast, the fact that a 3D environment can also be understood as a score may appear less self-evident. I would this like to explain my thoughts on this by way of a concrete example. When designing *Kilgore’s* 3D environment, I spent some time smoothing bumpy sections of the paths and ravines along which the avatars usually move, as otherwise the avatars would frequently get stuck behind these uneven patches and need to perform a jump to continue on their path, interrupting the flow of movement. Initially, I sought to avoid the gaps produced by uneven sections.

When rehearsing *Kilgore*, however, the following unexpected scenario occurred: at a certain point in the piece, one of the avatars has to move to a position that can only be reached by running through a long ravine. Furthermore, at this point in the piece a functionality is activated that causes objects to fall from the sky when the jump function is used. These objects produce feedback-like sounds when they land. During the rehearsals it became clear that I had not made this ravine smooth enough, which made the avatars get stuck and meant that they could only move forward by jumping. This triggered a large number of the falling objects and their feedback effects. What had initially been an oversight in developing the 3D landscape unexpectedly gave this particular part of the piece a special character of its own. There was no other part of the composition in which the mentioned feedback-producing objects were triggered so frequently. In this particular context, this design ‘flaw’ provided musical interest and became characteristic of the piece’s formal structure.

I have described this scenario in such detail because I consider it an excellent example of how the design of a landscape implicates direct – and, in this case, unforeseen – musical decisions. When looking at such an environment it its entirety, taking its musical characteristics and contingencies into consideration, the analogy of the environment as a score presents itself, in the sense of a legible record and temporal organisation of musical events. One key point in this analogy to a score is that the environment and its musical possibilities can be read by human beings, as for example an audiotape is also able to record and organise musical events in time, but does not offer these events in a form legible by humans.^4^ In a 3D environment, the temporal organisation is not determined as strictly as in traditional scores. As I have already explained above, however, every intervention in the environment’s topology also implicates musical decisions, especially decisions concerning temporal organisation.

## Composing with rules, situations and stimuli

Composition using gaming elements is characterised by the definition, in one way or another, of rules according to which the performers acts and according to which a technological environment behaves. In most cases, such rules relate to global design decisions. Thus a rule in the example mentioned in the previous section defines that, at a certain stage of the game, an object falls from the sky each time an avatar uses the jump function. This object generates feedback-like sounds upon impact. This rule relates to a formal section in which this principle applies and co-creates the musical process. This case marks a form of top-down design, as it defines a link between events that plays out regardless of the exact circumstances and details that trigger this function. In general, it can be said that rules only rarely offer ways of shaping the fine details of the musical realisation (Ciciliani & Lüneburg, 2018).^5^ Accordingly, when composing with rules, we often find ourselves confronted with the question of how to influence these subtle musical details.^6^

In my compositional work with gaming elements, I have found it increasingly necessary to adopt the performers’ experiential perspective rather than searching for a certain sound phenomenon for a selected point in time. The question guiding my compositions is thus not which sound event I want to occur exactly when, but rather: how can I create a situation in which the performers are motivated to perform a certain musical act? Thus I compose situations and stimuli rather than sounds. I try to create a situation that on the one hand corresponds to a precise musical idea, without being able to directly shape the sound events that may occur within said situation. On the other hand, the situation provides a number of affordances, which in their entirety aim to create an interesting and stimulating situation for the performers. Thus motivated, the performers’ actions convey the intended musical quality. In the context of the design of game spaces, game theorist Michael Nitsche refers to this phenomenon as *Attractors* or *Perceptual Opportunities* (2008)*.* He argues that ‘[…] spaces evoke narratives because the player is making sense of them in order to engage with them. Through a comprehension of signs and interaction with them, the player generates new meaning.’ (2008, p. 3).

In the given musical context I argue that in contrast to working with rules, composing situations and stimuli evokes detailed actions on behalf of the performers that can lead to corresponding musical events. Hence, contrary to rule designs, we can here speak of a bottom-up design. This designing of detail can be combined with the designing of rules, though, so the two approaches actually complement each other. Composing rules and stimuli is certainly not a principle restricted to work in 3D spaces. However, because of the virtual presence that performers are able to experience through their avatars, the detailed design of virtual affordances seems particularly appropriate here.

## Polyspatiality

The following situation is more or less the classic case in audiovisual concerts: the performers enter the stage, and as the playing starts, a wide-screen video projection appears above the musicians’ heads. For the rest of the performance, the audience remains glued to what is happening on the screen and almost forgets the performers present on stage. Situations such as this are not unusual, and there is nothing fundamentally problematic about them – there are many great audiovisual compositions in which such situations are created without compromising the piece’s artistic quality in the slightest. Nevertheless, I do ask myself why the visual space of the screen often dominates the events on the real space of the stage so strongly.

When working with 3D environments, where the performance takes place in both physical and virtual space, this particular constellation renders the question even more pressing: how can the relation between physical and virtual space be shaped, a direct link between these two levels established and the real space of the stage be made relevant for the experience as a whole? Is it possible to design a time-variant spatial, topological or architectural counterpoint? These questions require further investigation, which I have not been able to carry out systematically this far. Nevertheless, in the following I would like to formulate three observations and theses that can serve as a starting point for further analysis.

First, according to my observations, if the projected image is created as the result of interactions between the musicians on stage that the viewers are able to follow, the audience’s awareness is less likely to be completely absorbed by the video. This interaction means that the performers are more tangible and thus to a greater degree present as actors in the projection, which links the image more closely and directly to events on stage. In the case of compositions that use gaming elements, there is basically always a more or less direct level of interaction between the performers and the audiovisual setup. It thus seems logical to consciously design the way that the actions are translated between the two levels of projected and physical space. This can mean, for example, that the performers’ actions are related directly to the associated behaviour of their avatars. In other words, it can mean that the actions and their translations are transparent and legible.

Second, in my experience, a single projection screen in the performance setup has particularly strong absorbing effect. I describe this setting as a *cinematic* setup, in the sense that viewers are focused completely on the screen and block out their environment, similarly to a cinema screening. Interestingly, however, using two different projections already breaks the screen’s pull. I compare this with an ‘installative’ situation. Because viewers’ visual attention is no longer drawn to a single screen, we are dealing with a setup that is thus not simply distributed across two screens but includes and makes the audience more aware of the whole of the physical space. In other words, while a single screen remains a singular unquestioned attractor, two or more screens articulate a space that also includes the performers and the audience (Petersen, 2015). I have observed that the events and performers on stage are better integrated into the whole when two or more projections are involved.

Third, a further means of emphasising the stage more strongly is the time-variant use of light. I have used this several times in projects with a single projection screen, with the light on the stage mirroring colour changes in the projection, for example. This made it seem as if the projection were ‘spilling over’ onto the stage and into the space. Measures such as these are able to reduce the screen’s visual exclusivity, drawing attention back to the performance in the physical space and thus creating a more balanced audiovisual experience overall.

## Mapping

The following section starts with a somewhat more technical consideration of the way streams of data are generated by the performers’ actions and can be used subsequently for interactive audiovisual designs. The performers’ movement through an environment via their avatars takes place through the so-called input data they generate. In the case of a normal game controller, this usually happens via a joystick that generates data on movement along the X, Y and Z axes and thus describes the direction of movement. In research on Human–Computer Interaction (HCI), the way that data are allocated to the parameters of a digital system is referred to as ‘mapping’. These allocations can be described using the ratio between the input and output signals.

The simplest configuration is one-to-one mapping. Here, a single input signal is allocated to a single parameter. For example, if I move a control and this changes only the volume of the given signal, we are dealing with one-to-one mapping. One-to-many mapping describes when an input signal affects several parameters. For example, we could imagine the same input signal changing not just the volume, but a filter setting, so that the sound becomes brighter as it grows in amplitude.

Besides one-to-one and one-to-many we also have many-to-one mapping, which is present for example in wind instruments, where changes in both blowing pressure and finger position can alter the pitch. Accordingly, several input data influence the same musical parameter.

Finally, there is also many-to-many mapping, which covers a large number of possible combinations. Another analogy to a traditional instrument serves as an example here: with string instruments, for example, a finely coordinated interaction of bow speed, bow pressure and the bow’s position on the string produces a certain timbre that affects both various sound parameters and the volume. What is distinctive here is that the individual input data can no longer be attributed directly to a single change in the sound, but that the complex process of precisely coordinating the parameters with one another leads to changes in the overall sound produced.

In the context of work with 3D environments, I will now describe a two-to-many scenario.^7^ Above, as an example of one-to-many mapping I mentioned that an input signal can be allocated to both the volume and filtering of a sound source. This could be expanded as desired, of course. But if the input signal is mapped in the same linear manner to a number of parameters, the result often feels one-dimensional and inflexible, despite this distribution across parameters. It would be preferable for the input signal to be conveyed not linearly, but in dynamically shifting weightings that diverge slightly from one another and shift dynamically depending on context.

In the following examples, I am describing a situation where the movement of an avatar in 3D space is controlled by supplying two input signals, one for front and back, and one for left and right, as it is e.g. often the case with computer games. The scenario could just as well be extended to a situation where movement is controlled along all three dimensions, which would not fundamentally alter the described phenomenon. I am therefore confining the example to two dimensions for the sake of simplicity.

**Figure 5:**
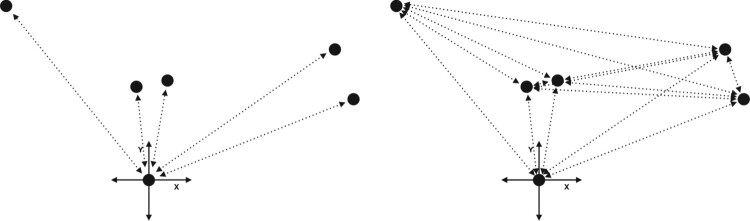
Examples of two-to-many mappings. On the left, the relations between the avatar’s position and five objects in the environment are measured, resulting in a 2 in/5 out scenario; on the right, the distances between the objects are measured, resulting in a 2 in/15 out scenario.

If we start from the navigation of the avatar via an X and a Y axis and use two input signals, within a 3D environment we are able to derive a far greater number of parameters by measuring the avatar’s position in relation to various objects or positions within the 3D environment. Figure 5 shows such a situation: an avatar is moving in the X-Y plane and its distance to five objects or positions in the space is being measured. Accordingly, one single movement is used to define five different data streams. As we are dealing with a situation in which the performers are virtually present through their avatars, the changes in these five data streams takes place in an intuitively comprehensible way. This is relevant if the input data are allocated to parameters including musical options, for example. For this reason, the design of such a mapping can be understood as part of the composition of affordances and stimuli described above.

This distribution of data across parameters is even more extreme if we measure the distance to objects that are themselves moving or that can be moved by interacting with them. If we also measure the relative distances between these objects to generate input data, in the case of five objects we are able to generate no fewer than 15 signals. This is depicted in Figure 5. It is interesting to note here that not only data describing the avatar’s relation to the world are generated, but also data that reflect a state in the environment, in this case the relation of the moveable objects to one another. I once created such a situation in a model where the avatar interacted with a large number of moveable virtual balls, all of which produced a pitch that changed when the avatar touched them. The balls’ movement was the result of their interaction with the avatar. The data gained by measuring the distance between the various balls could thus be understood as a lasting effect of the previous action. When implemented, this can anchor the performers more strongly in the virtual environment, as they are able to feel the consequences of their actions.

What is striking about the described scenarios is that the various input data are generated directly from the avatars’ movement and interaction, which themselves are steered only through two input channels. Accordingly, the many signals produced still change in an intuitively comprehensible way, rendering them very interesting for audiovisual interactions.

## Brief conclusion

Work with 3D environments offers a range of musical potential and various different creative possibilities: the conscious composition of space and movement, the representation of the performers in the virtual space while being present in the real space at the same time, the use of the 3D environment as both instrument and score, the use of polyspatiality, and not least the interaction and mapping of performers’ actions in the virtual environment. This sheds light on many areas of the field that still require further artistic and theoretical enquiry.

## Footnotes

1 Funded by the FWF (Österreichischer Wissenschaftsfond) as part of the PEEK programme as AR364-G24, running 2016–2020, head of project: Marko Ciciliani.

2 This text is based not only on my more substantial composition *Kilgore*, but also on individual 3D studies that I produced as part of my work on this piece. The text also draws upon further similar works from the GAPPP research project, in particular *Game Over* and *Terrain Study* by Christof Ressi (2018) and *Trois machins de la grâce aimante* by Rob Hamilton. Translation: Dr. Margaret Hiley.

3 Cf. https://vimeo.com/303861609/03260f67ba and http://vimeo.com/303861838/1d2eb2889b (last accessed 25 November 2018).

4 During the rehearsal process, the musicians repeatedly used the full view of the 3D landscape reproduced in Figure 3 to prepare for the performance of the piece. In this situation, it did indeed function as a score in the sense described above.

5 This is the case particularly when the rules govern the performers’ behaviour. When rules are applied in algorithmic processes, it is easier to influence the fine details.

6 Rule-based works usually dispense with traditionally notated scores as most of them are conceived as open-form compositions. For this reason, it is normally not possible to notate musical details.

7 The following description is indebted to Rob Hamilton, who employed various forms of ‘few-to-many’ mappings in his work with 3D environments.
